# Oil Accumulation in Transgenic Potato Tubers Alters Starch Quality and Nutritional Profile

**DOI:** 10.3389/fpls.2017.00554

**Published:** 2017-04-12

**Authors:** Madeline Mitchell, Jenifer Pritchard, Shoko Okada, Oscar Larroque, Dina Yulia, Filomena Pettolino, Nicolas Szydlowski, Surinder Singh, Qing Liu, Jean-Philippe Ral

**Affiliations:** ^1^Commonwealth Scientific and Industrial Research OrganisationCanberra, ACT, Australia; ^2^Univ. Lille, CNRS, USR 3290 - MSAP - Miniaturisation pour la Synthèse l'Analyse et la ProtéomiqueLille, France

**Keywords:** potato, carbon partitioning, nitrogen partitioning, triacyglycerol, starch, metabolic engineering

## Abstract

Plant storage compounds such as starch and lipids are important for human and animal nutrition as well as industry. There is interest in diverting some of the carbon stored in starch-rich organs (leaves, tubers, and cereal grains) into lipids in order to improve the energy density or nutritional properties of crops as well as providing new sources of feedstocks for food and manufacturing. Previously, we generated transgenic potato plants that accumulate up to 3.3% triacylglycerol (TAG) by dry weight in the tubers, which also led to changes in starch content, starch granule morphology and soluble sugar content. The aim of this study was to investigate how TAG accumulation affects the nutritional and processing properties of high oil potatoes with a particular focus on starch structure, physical and chemical properties. Overall, TAG accumulation was correlated with increased energy density, total nitrogen, amino acids, organic acids and inorganic phosphate, which could be of potential nutritional benefit. However, TAG accumulation had negative effects on starch quality as well as quantity. Starch from high oil potatoes had lower amylose and phosphate content, reduced peak viscosity and higher gelatinization temperature. Interestingly, starch pasting properties were disproportionately affected in lines accumulating the highest levels of TAG (>2.5%) compared to those accumulating only moderate levels (0.2–1.6%). These results indicate that optimized engineering of specialized crops for food, feed, fuel and chemical industries requires careful selection of traits, and an appropriate level of transgene expression, as well as a better understanding of starch structure and carbon partitioning in plant storage organs.

## Introduction

Plants are important sources of starch and lipids (oil) for food and industry. Starch is the primary storage compound in leaves, cereal grains, and tubers while lipids are a major component of seeds such as sunflower and oil palm. As well as contributing significantly to human caloric intake, starch granules have a complex molecular structure that has led to many applications in food processing and industry (Jobling, 2004). Lipids are also a major source of calories and demand for plant oil is increasing due to changing diets, a growing population and increased non-food applications (FAO, 2003). Current crops produce either starch or lipids but recent metabolic engineering has raised the possibility of generating crops capable of producing both starch and lipids.

Increasing the lipid content of starch-rich organs could improve the nutritional value or energy density of crops as well as increase lipid production for food, feed, fuel, and chemical industries. It may even be possible to generate crops that provide both a conventional grain harvest as well as a biomass harvest for oil. Significant progress has already been made in the production of biomass oil crops with TAG accumulating to 2 and 35% (DW, dry weight basis) in the vegetative tissue of sugar cane and tobacco, respectively (Zale et al., 2016; Vanhercke et al., 2017). Alternative metabolic engineering approaches focusing on increasing the lipid content of storage organs such as potato tubers have also proved successful, with TAG content ranging from 0.3 to 1% DW (Klaus et al., 2004; Hofvander et al., 2016).

However, in engineered sugarcane, tobacco and potato, TAG content was negatively correlated with starch content suggesting that lipid accumulation affects carbon partitioning in both photosynthetic and storage organs. This has implications for plant growth as well as potential biotechnological applications. Plants with low starch may not grow as well as wild-type plants, especially in short days (for example, Caspar et al., 1985; Lin et al., 1988; Huber and Hanson, 1992), while reduced starch yield or quality might affect the types of applications possible for a particular crop (Slattery et al., 2000; Zeeman et al., 2010; Sonnewald and Kossmann, 2013). Changes to other metabolite levels may also affect the nutritional value of the crop. Previously, our group developed transgenic potato lines with tubers accumulating over 100-fold more TAG than wild-type, or up to 3.3% TAG by dry weight, and preliminary assessment indicated that starch structure and accumulation were negatively affected (Liu et al., 2017).

In light of this, the aim of the present study was to investigate the effects of TAG accumulation on starch structure as well as the nutritional and processing properties of the transgenic potatoes. TAG accumulation was correlated with changes to starch granule composition and structure as well as chemical and physical properties. The high oil potato lines thus provide an interesting system to investigate the molecular regulation of starch synthesis and the relationship between starch structure and physical properties. An understanding of carbon partitioning in plant storage organs and its effect on starch structure will guide future attempts to increase lipid yield while maintaining starch and carbohydrate quality. This study also highlights the importance of selecting appropriate traits and transgene expression in order to optimize the production of specialized crops for food, feed, fuel, and chemical industries.

## Methods

### Plant material and growth conditions

Wild-type (*n* = 4) and transgenic potato plants (*Solanum tuberosum* L., cv. Atlantic) were grown in the glasshouse and potatoes were harvested at maturity as described in Liu et al. (2017). Eight independent transgenic high oil lines were selected with varying levels of TAG accumulation and expression of the transgenes *WRINKLED1* (*WRI1*, lipid-related transcription factor from *Arabidopsis thaliana*), *diacylglycerol acyltransferase 1* (*DGAT1*, TAG biosynthetic enzyme from *A. thaliana*) and *oleosin* (lipid packaging protein from *Sesamum indicum*). Tissue from potatoes was freeze dried for 72 h prior to lipid, starch, soluble sugar and metabolite analysis. Fresh tuber tissue was used for starch extraction.

### Whole potato macronutrient analysis

Total carbon and nitrogen in freeze-dried powderized potato (potato flour) was determined using a Europa 20–20 isotope ratio mass spectrometer with an ANCA preparation system, comprising a combustion and reduction tube operating at 1,000°C and 600°C, respectively. Potato TAG was quantified by chloroform/methanol/water extraction, thin layer chromatography separation, fatty acid methylation and gas chromatography as described in Liu et al. (2017). Measurements of the total starch and sugar content of potato flour were as described in Liu et al. (2017). Cellulose was measured using the method of Updegraff (1969) with an additional digestion of the starch using α-amylase and amyloglucosidase (Total Starch Kit, Megazyme, Wicklow, Ireland). Soluble protein was extracted in buffer (Tris pH 7.5, 10 mM MgCl_2_) and quantified by Bradford assay (Bradford, 1976).

### Metabolite analysis

Metabolite extraction, derivatization and gas chromatography mass spectrometry (GC-MS) analysis were conducted based on Roessner et al. (2000) with the following modifications. Freeze-dried tuber tissue (10–20 mg) was homogenized with 200 μl of methanol. Then 300 μl of 0.1 mg ml^−1^ ribitol (Sigma-Aldrich, St. Louis, MO, USA) in H_2_O was added and samples were again homogenized. After centrifugation at 16,100 × g for 5 min, 30 μl of the supernatant was dried down and derivatized at 37°C for 90 min with 15 μl of 20 mg ml^−1^ methoxyamine hydrochloride (Sigma-Aldrich) in pyridine (ACS reagent grade, Sigma-Aldrich). Following derivatization, 15 μl of N-methyl-N-(trimethylsilyl)-trifluoroacetamide (GC derivatization grade, Sigma-Aldrich) was added to each sample and further incubated at 37°C for 30 min. Aliquots (25 μl) of derivatized sample were transferred to 2 ml GC sample vials along with 5 μl of n-alkane standard mix (0.029% [v/v] n-dodecane, 0.029% [v/v] n-hexadecane, 0.029% [w/v] n-eicosane, 0.029% [w/v] n-heneicosane, 0.029% [w/v] n-docosane, 0.029% [w/v] n-pentacosane, 0.029% [w/v] n-octacosane), and left to equilibrate for 4 h. The samples were analyzed by GC-MS on a DB5MS column with a 10 m integrated guard column (0.25 mm internal diameter, 0.25 μm film thickness, 30 m length; Agilent Technologies, Santa Clara, CA, USA) fitted to an Agilent 7890A gas chromatograph with a 5975C inert MSD quadrupole detector (Agilent Technologies). Samples (1 μl) were injected at a 10:1 split into the inlet that was heated to 230°C with helium as the carrier gas with a total flow of 24 ml min^−1^, septum purge flow of 3 ml min^−1^, and a split flow of 10 ml min^−1^. The column gas flow was set at 1 ml min^−1^. The oven temperature was as follows: the initial temperature of 70°C was held for 1 min, then increased to 320°C at 15°C min^−1^, and held at 320°C for 3 min. The MSD transfer line was heated to 250°C, MS source at 230°C and MS quadrupole at 150°C. The electron impact ionization energy was 70 eV and the MS detector was run in full-scan mode with an m/z range between 35 and 550 and 2.83 scans s^−1^. MetabolomeExpress (Carroll et al., 2010) was used to process raw GC-MS data and conduct statistical analysis including orthogonal partial least squares analysis.

### Starch isolation and physical and chemical characterization

Starch was isolated as described in Liu et al. (2017). Starch granule size distributions were determined using a laser diffraction particle size analyzer (Mastersizer 2000, Malvern Instruments, Malvern, United Kingdom). Apparent amylose content was determined by colorimetric assay using iodine solution (Glaring et al., 2006) on non-defatted starch. Chain-length distribution of starch granules was analyzed by fluorophore-assisted carbohydrate electrophoresis as described by Morell et al. (1998). C3- and C6-phosphoesters in the starch were determined by fluorescence-assisted capillary electrophoresis as described by Verbeke et al. (2016).

### Pasting properties of starch by rapid visco analyzer

Pasting properties of isolated starch were determined using a Rapid Visco Analyzer RVA-4SA (Perten Instruments, Sydney, Australia). Starch samples (2 g) and deionized water (25 ml) were weighed directly into a test container and mixed well using a plastic paddle before loading on the instrument. Starch suspensions were stirred at 960 rpm for 10 s after which stirring was reduced to 160 rpm for the remainder of the experiment. Samples were equilibrated at 50°C for 2 min, heated to 95°C at 7.5°C min^−1^, held at 95°C for 4 min, cooled to 50°C at 11.25°C min^−1^ and then held for 4 min at 50°C. Peak viscosity, hold viscosity (end of maximum temperature holding phase), final viscosity and pasting temperature were recorded using the Thermocline software provided with the instrument.

### Hot stage microscopy and swelling test

A T95 series temperature controller coupled with a temperature controlled stage and Linksys32 software (Linkam Scientific, Tadworth, UK) fitted to a Leica DM6 B microscope with CTR6 LED controller and LASX software (Leica Microsystems, Wetzlar, Germany) was used to analyse loss of birefringence of isolated starch granules under polarized light during heating. Aliquots (2 μl) of homogenous slurries of starch in water (25 mg ml^−1^) were mounted onto StarFrost silane coated slides (ProSciTech, Thuringowa, QLD, Australia). The coverslip was sealed with nail polish to prevent streaming of starch granules during heating. Hot stage temperature ramp rate was 4°C per minute to a maximum of 90°C. Images were taken at 15 s intervals over 17 min. Intensity profiles created in LASX software were exported to Microsoft Excel. The initial intensity was set at 100% and profile temperatures (95, 50, and 5% of initial intensity) were calculated assuming a linear loss of intensity. Only wild-type and starch from the highest TAG lines were analyzed as these were expected to reveal the greatest differences. Starch swelling power was determined using the method of Konik-Rose et al. (2001).

### Thermal properties of starch by differential scanning calorimetry

Thermal properties of isolated potato starches were determined using a DSC 8000 with 96 well plate format (Perkin Elmer, Waltham, MA, USA). Starch/water mixtures (1:3 [w/v], using average starch moisture content of 15%) were vortexed vigorously and incubated at 4°C for 16 h. Triplicate aliquots (50–55 μl) were weighed into stainless steel capsules with rubber O-rings. The program heated from an initial temperature of 20°C to 140°C at 10°C min^−1^ then cooled to 20°C at 20°C min^−1^ with a nitrogen gas flow of 20 ml min^−1^.

### Imaging of isolated starch granules

Isolated starch granules were stained with iodine and imaged using an Axio Imager light microscope (Ziess, Jena, Germany). Isolated granules were also sputter-coated with gold and imaged using a Zeiss EVO LS 15 scanning electron microscope at 5 kV. Starch granules were observed *in situ* by light microscopy of fresh, unstained sections (Axio Imager).

## Results

### Lipid accumulation in transgenic potato lines alters macronutrient profiles

**Figure 1:**
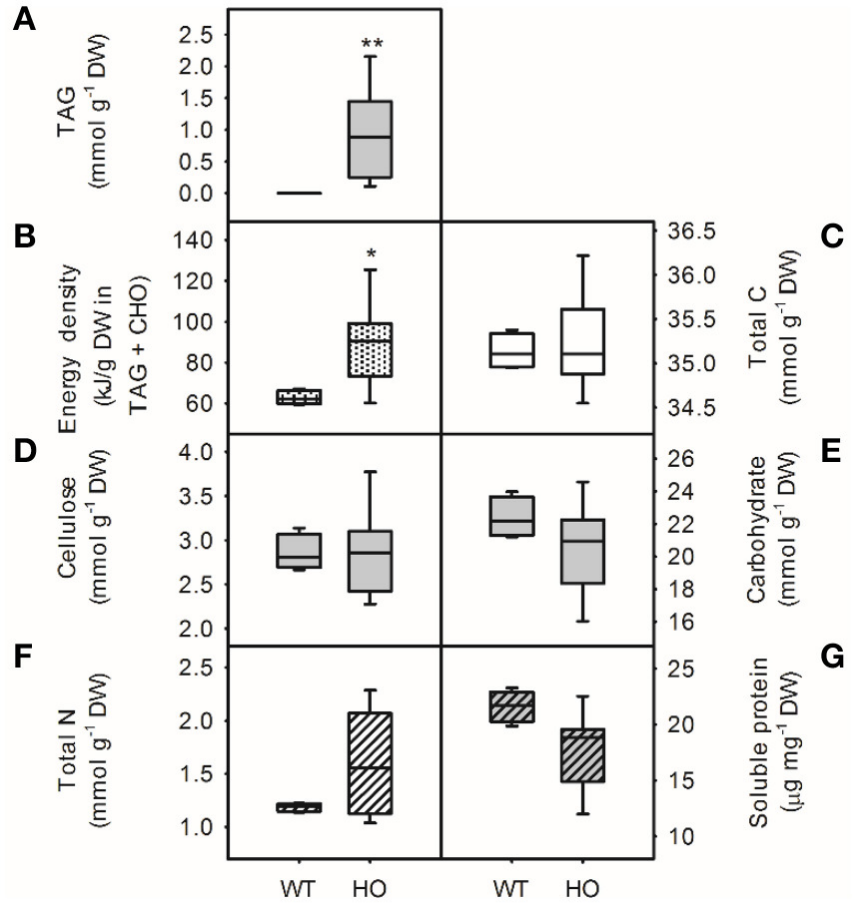
**Energy density and nutritional profile of wild-type (WT) and transgenic high oil (HO) potato tubers**. Energy density **(B)** calculated from total energy stored in triacylglycerol (TAG) and carbohydrates. Potato total carbon **(C)** and amount of carbon found in lipids (TAG, **A**); fiber (cellulose, **D**); carbohydrates (starch and soluble sugars, **E**); total nitrogen **(F)** and soluble protein **(G)**. Asterisks indicate significant differences between WT and HO (*t*-test: ^*^p < 0.05; ^**^p < 0.01). See Supplemental Table 1 for correlation matrix.

The effect of TAG accumulation on the nutritional profile of high oil potatoes was investigated (Figure 1). TAG content was significantly higher (*t*-test, *p* < 0.01) than wild-type levels (<0.05% DW) in all eight independent transgenic potato lines but varied widely from 0.17% DW to 3.35% DW (Figure 1A). The energy density of tubers also increased up to two-fold in high oil potato lines (55.4–117.8 kJ g^−1^ DW) compared to wild-type (54.4–61.3 kJ g^−1^ DW; *t*-test, *p* < 0.05) although the total carbon content was unchanged (Figures 1B,C). The mean cellulose (fiber) content of tubers was the same for wild-type and high oil lines (8.6% DW; Figure 1D). Despite these changes to carbon partitioning, carbohydrates (starch and soluble sugars) remained the major carbon reserves in high oil potatoes (approximately 60% of the total carbon). There was no difference in the total carbohydrate content of wild-type and high oil lines (Figure 1E), although TAG had previously been correlated with decreased starch and increased soluble sugars (Liu et al., 2017).

Overall, high oil potato appeared to have increased total nitrogen and decreased soluble protein content compared to wild-type potatoes (Figures 1F,G, respectively); however, due to the large variation across high oil lines, these differences were not statistically significant. There was a significant positive correlation between total nitrogen and potato TAG content (*r* = 0.94, *p* < 0.01; Supplemental Table 1) but there was no significant correlation between soluble protein and TAG content. Quantification of the major 40 kDa band suggested a 25% reduction in the major storage protein patatin in high oil potatoes (Supplemental Figure 1). Significant accumulation of 17 free amino acids in high oil potatoes compared to wild-type was also observed, which would contribute to the observed positive relationship between TAG and total nitrogen (Table 1).

**Table 1:** **Differentially accumulated metabolites in wild-type (WT) and transgenic high oil (HO) potato tubers**.

**Metabolites more abundant in WT**		**FC (WT/HO)**
	Citrate	2.23
	L-asparagine	1.29
**Metabolites more abundant in HO**		**FC (HO/WT)**
Sugars	D-Fructose	100
	D-Galactose	100
	D-Glucose	100
	D-Mannose	14.3
	Sucrose	2.44
Sugar phosphates	Fructose 6-phosphate	3.70
	Glucose 6-phosphate	3.70
	Mannose 6-phosphate	4.17
Amino acids	L-Alanine	16.7
	γ-Aminobutyric acid	1.35
	L-Aspartic acid	1.79
	Glycine	2.70
	L-Glutamine	4.76
	L-Homoserine	16.7
	L-Isoleucine	12.5
	L-Leucine	4.35
	L-Lysine	7.69
	L-Methionine	7.14
	L-Phenylalanine	20.0
	L-Proline	20.0
	L-Serine	2.72
	L-Threonine	5.88
	L-Tryptophan	16.7
	L-Tyrosine	33.3
	L-Valine	11.1
Amino acid derivative	Agmatine	1.75
	Putrescine	1.67
	Pyroglutamic acid	4.76
Sugar alcohols	Glycerol	1.23
	Mannitol	100
	Sorbitol	100
Carboxylic acids	Fumaric acid	2.70
	L-Malic acid	10.0
	Nicotinic acid	1.49
Hydroxy acid	Ascorbic acid	5.56
	Glyceric acid	1.75
	Threonic acid	1.79
*Other*	Phosphate	3.57

*Metabolites shown were significantly more abundant (p < 0.05) in either wild-type (WT, n = 4) or high oil (HO, n = 8) potato lines based on triplicate measurements for each line. Fold-change (FC) refers to the ratio of signal intensities for each metabolite*.

Overall, the increase in total nitrogen but not total carbon in transgenic lines led to a significant negative correlation between tuber TAG content and the carbon-to-nitrogen ratio (*r* = −0.92, *p* < 0.01). Correlation coefficients between TAG and the other carbon- and nitrogen-containing components are shown in Supplemental Table 1.

### Oil accumulation is associated with changes to other metabolites

Metabolites were analyzed by GC-MS to further investigate the nutritional profile of wild-type and high oil potatoes (Table 1). A total of 40 differentially accumulated metabolites were positively identified based on retention times and mass-to-charge ratios. Of these, 38 metabolites were more abundant in high oil potatoes and two were more abundant in wild-type. Of the additional metabolites that were detected but not positively identified, approximately 60% were more abundant in high oil potatoes, 3% were more abundant in wild-type and the remaining 37% were not differentially accumulated (Supplemental Dataset 2).

Changes to metabolite abundance were consistent across classes of compounds with the majority of changes occurring in monosaccharides and disaccharides, amino acids and their derivatives. Five sugars, three sugar phosphates and three sugar alcohols were all more abundant in high oil potatoes, which was consistent with colorimetric total water-soluble sugar measurements. Seventeen amino acids and three amino acid derivatives were more abundant in high oil potatoes, which was consistent with total nitrogen and soluble protein measurements. Six organic acids were more abundant in high oil potatoes, including two intermediates of the citric acid cycle and the nutrients ascorbic acid and nicotinic acid (niacin). Inorganic phosphate was also more abundant in high oil potatoes. The two metabolites that were more abundant in wild-type potatoes consisted of one citric acid cycle intermediate (citrate) and one amino acid (asparagine). Orthogonal partial least squares analysis of metabolite profiles revealed that lines accumulating the highest levels of TAG (>2.5%) clustered separately to those accumulating lower levels of TAG (0.2–1.6%) and to wild-type lines (Supplemental Figure 2).

### Starch from high oil potatoes has altered molecular structure

**Figure 2:**
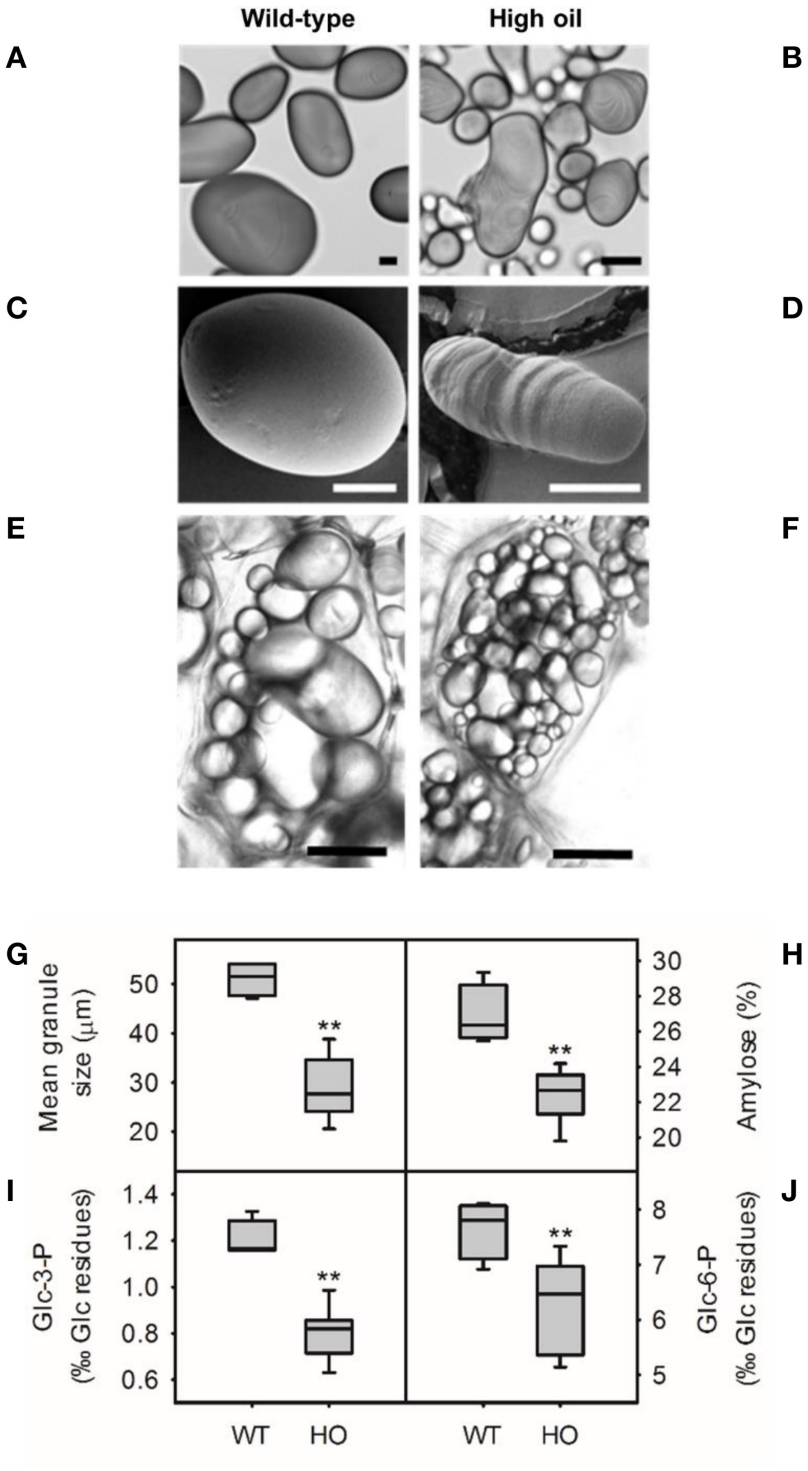
**Starch granule morphology from wild-type (WT) and transgenic high oil (HO) potato tubers**. Light micrographs of isolated starch stained with iodine (**A,B**, scale bars = 10 μm) and scanning electron micrographs of isolated starch granules (**C,D**, scale bars = 10 μm) from WT **(A,C)** and HO **(B,D)** lines. Representative images of starch granules *in situ* in fresh unstained sections from WT **(E)** and HO **(F)** potatoes (scale bars = 30 μm). Starch granule size **(G)**, apparent amylose content **(H)** and phosphorylation of glucose residues at C3- **(I)** and C6- **(J)** positions. Asterisks indicate significant differences between WT and HO (*t*-test: ^**^p < 0.01).

Changes to granule morphology were previously observed in starch from high oil potatoes (Liu et al., 2017). Starch was isolated from wild-type and high oil potatoes to investigate this in more detail and to determine whether changes to chemical structure accompanied alterations in morphology (Figure 2). Starch granules from wild-type potatoes were large and uniformly elliptical in shape with a mean granule diameter of 51 μm (Figures 2A,C,G). Starch granules from high oil potatoes were smaller (20.6–38.8 μm diameter), more elongated and noticeably misshapen with strong striation patterns and double hila observed even under a light microscope (Figures 2B,D,G). The surface of high oil starch granules was often pitted (Figure 2D) whereas the surface of wild-type starch granules was smooth (Figure 2C). Starch granules were also observed *in situ* and qualitative observations suggested that starch granules were more numerous in high oil compared to wild-type cells and that cell size also appeared to be reduced (Figures 2E,F).

Starch structure at the molecular level was investigated to determine whether this could be causing the observed changes in starch morphology. The apparent amylose content of starch was reduced by up to one quarter in high oil potato lines (Figure 2H) although analysis of starch-bound proteins by SDS-PAGE found no relationship between amylose content and granule-bound starch synthase expression (GBSS; Supplemental Figure 1). The mean amylose content of wild-type starch was 26.9% whereas the amylose content of high oil potato starch ranged from 19.8 to 24.3%. Amylose content was negatively correlated with TAG accumulation (*r* = −0.79, *p* < 0.01) and positively correlated with granule size (*r* = 0.80, *p* < 0.01; Supplemental Table 2). The phosphate content of starch was also reduced in high oil potato lines (Figures 2I,J). The proportion of glucose residues phosphorylated at the C3- position ranged from 0.63 to 0.99%0 in high oil potato starch compared to a mean of 1.2%0 in wild-type starch. Similarly, the proportion of glucose residues phosphorylated at the C6- position ranged from 5.1 to 7.3%0 in high oil potato starch compared to a mean of 7.7%0 in wild-type starch.

**Figure 3:**
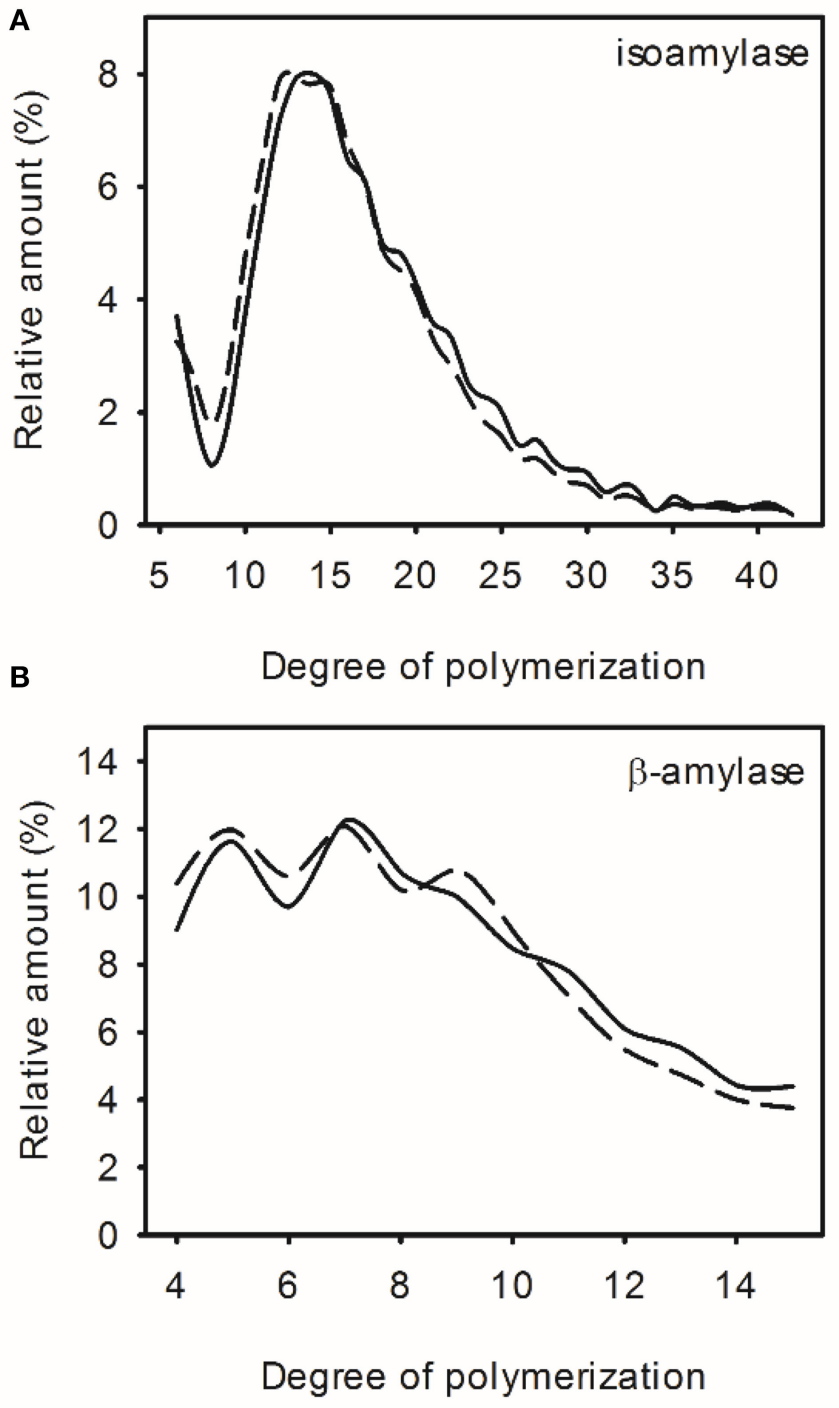
**Amylopectin structure in wild-type (WT) and transgenic high oil (HO) potatoes**. Chain length distribution of amylopectin from WT (solid line) or the highest oil potato (dashed line) digested with isoamylase **(A)** or β-amylase **(B)**. See Supplemental Figure 3 for chain length distributions of all lines. See Supplemental Table 2 for correlation matrix of triacylglycerol content, starch chemical and physical properties.

The amylopectin chain length distribution was also analyzed but differences in amylopectin branching were mostly small and inconsistent (<1% difference in individual chain abundance) and within the variation observed between wild-type replicates (Figures 3A,B; Supplemental Figure 1).

### Starch from high oil potatoes has altered chemical and physical properties

**Figure 4:**
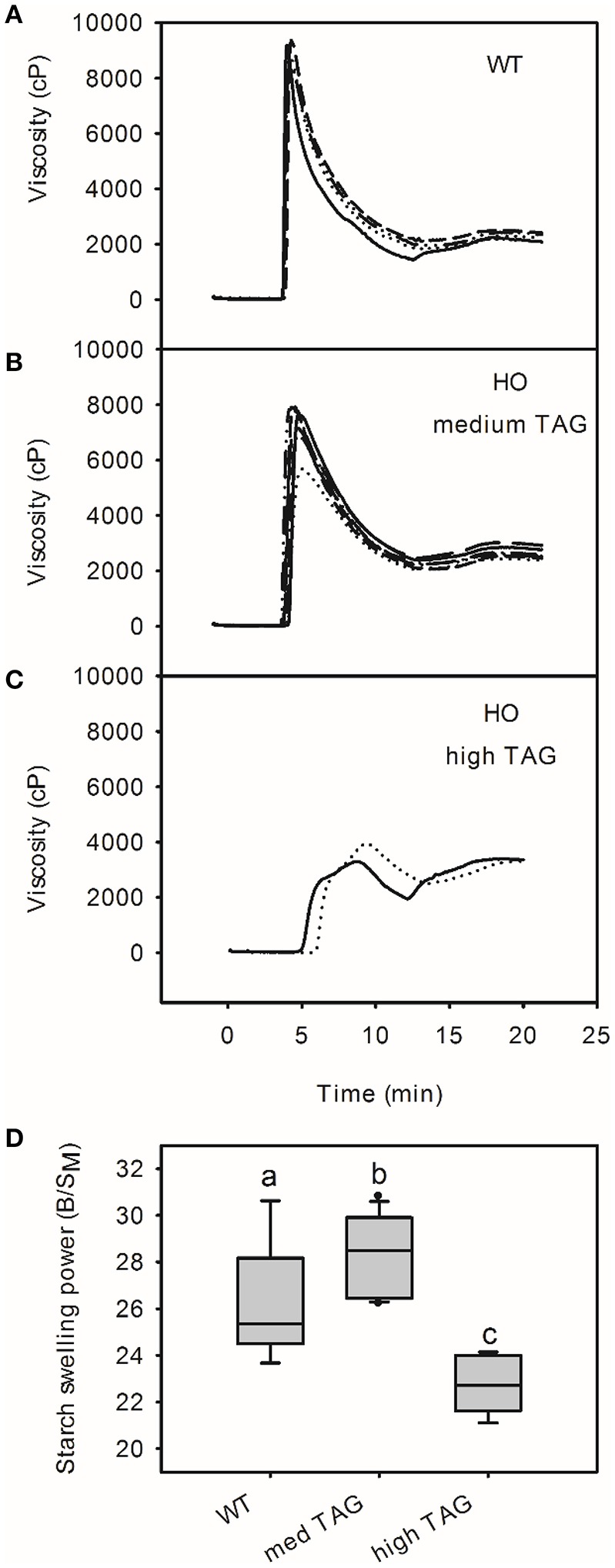
**Pasting profiles of starch from wild-type (WT; A)** and transgenic high oil (HO; **B,C**) potatoes by rapid visco analyzer. Starch pasting profiles from potatoes accumulating moderate levels of triacylglycerol (TAG; 0.17–1.63% DW; **B**) are shown separately to profiles from potatoes accumulating high levels of TAG (2.45–3.35% DW; **C**). Profiles from individual potato lines are shown using different lines. The swelling power of starch **(D)** is shown with letters indicating statistically significant differences (one way ANOVA). See Supplemental Table 2 for correlation matrix of triacylglycerol content and starch pasting properties.

The pasting profiles of isolated starch were determined using a rapid visco analyzer to investigate the effects of altered starch granule morphology and composition on its physical properties (Figure 4). The pasting profiles of starch from six of the eight high oil lines were similar in shape to those of the wild-type with peak viscosity at around 5 min (70–76°C). In contrast, the two transgenic lines accumulating the highest levels of TAG had starch with a peak at both 5 and 9 min (95°C). Starch from all high oil lines had lower peak viscosity than wild-type starch (mean values of 6,307 and 8,881 cP, respectively; *t*-test, *p* < 0.05) as well as higher hold viscosities (mean values of 2,234 and 1,834 cP, respectively; *t*-test, *p* < 0.05) and final viscosities (mean values of 2,784 and 2,271 Cp, respectively; *t*-test, *p* < 0.05). There was no significant difference in the pasting temperature between wild-type and high oil lines (50.13°C and 50.11°C, respectively; Figure 4). Correlation analysis identified strong correlations between starch granule size and peak viscosity (*r* = 0.78, *p* < 0.01), hold viscosity (*r* = −0.72, *p* < 0.01) and final viscosity (*r* = −0.70, *p* < 0.01; Supplemental Table 2). The amylose and phosphate contents of the starch (phosphorylation of glucose residues at C3- and C6- positions) were also correlated with peak (*r* = 0.79, *p* < 0.01; *r* = 0.76, *p* < 0.01, respectively) and final viscosities (*r* = −0.63, *p* < 0.05 for both amylose and phosphate; Supplemental Table 2).

The swelling power of “medium TAG” potato starch was slightly higher than that of wild-type starch (mean B/S_M_ values of 26.3 and 27.9, respectively; Figure 4D). In contrast, starch from lines accumulating the highest levels of TAG (“high TAG”) showed significantly reduced swelling power (mean of 22.7). This was consistent with the threshold effect observed in RVA profiles.

**Figure 5:**
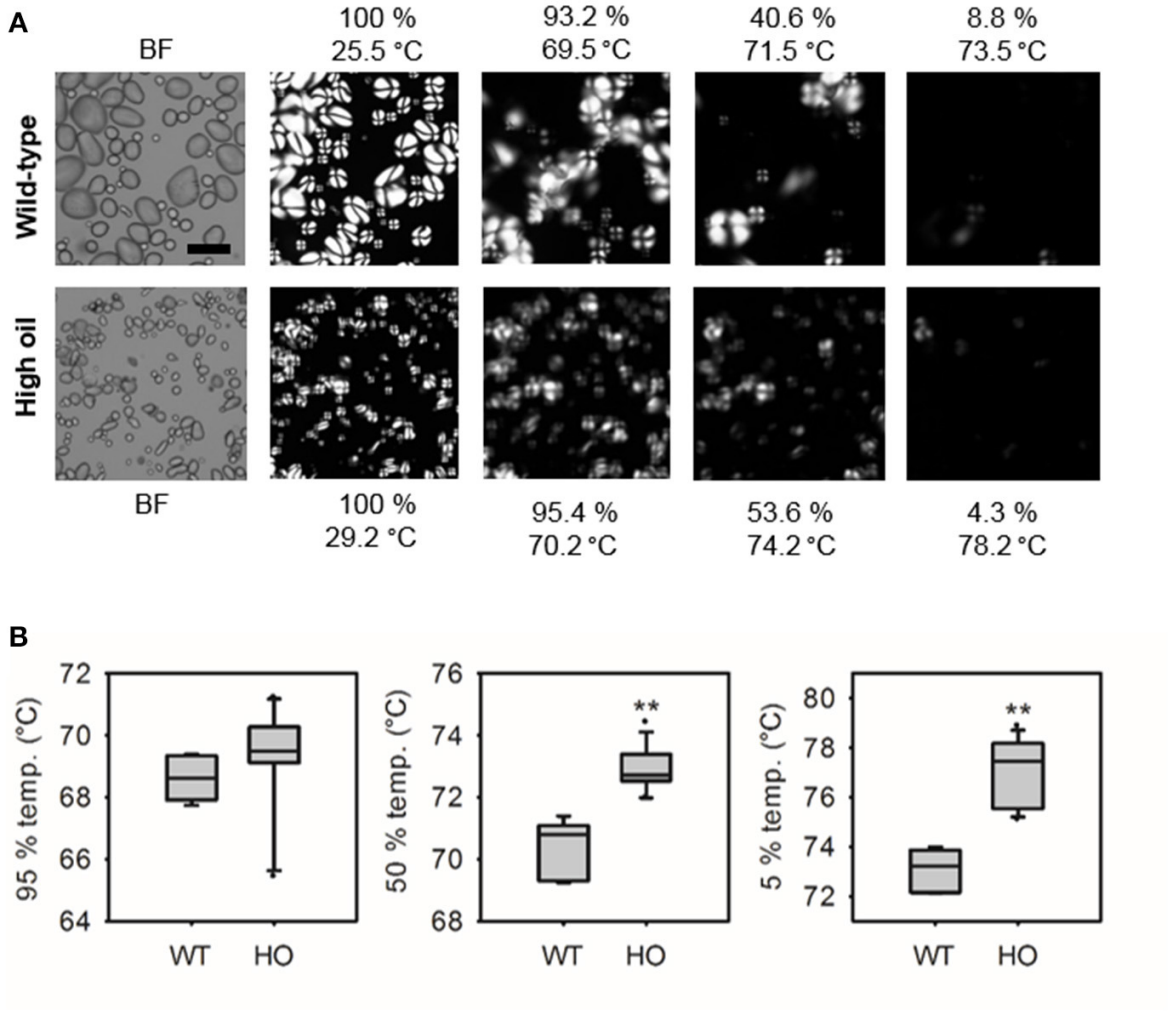
**Loss of birefringence of starch from wild-type (WT) and transgenic high oil (HO) potatoes during heating**. Representative brightfield (BF) images of starch prior to heating and images of starch birefringence at different temperatures using hot stage microscopy **(A)**. Quantification of loss of birefringence during heating **(B)**. Temperatures at which birefringence was 95, 50, and 5% of its initial intensity were calculated. Asterisks indicate significant differences between WT and HO (*t*-test, ^**^p < 0.01).

Gelatinization of starch from wild-type and the “high TAG” lines with distinct RVA profiles was further investigated using hot stage microscopy (Figure 5). When heated, starch from high oil potatoes retained some level of birefringence for longer than starch from wild-type lines (Figure 5A). While starch from high oil potato lines began to lose birefringence (95% temp.) at the same temperature as wild-type starch, high oil potato starch reached 50 and 5% of the initial birefringence intensity at significantly higher temperatures than wild-type starch (Figure 5B). Qualitative differences in the starch slurries and gels were also noted. Starch from lines accumulating the highest levels of TAG produced slurries that were frothy and formed cloudy gels whereas starch from wild-type and medium TAG lines produced clear slurries and gels.

**Figure 6:**
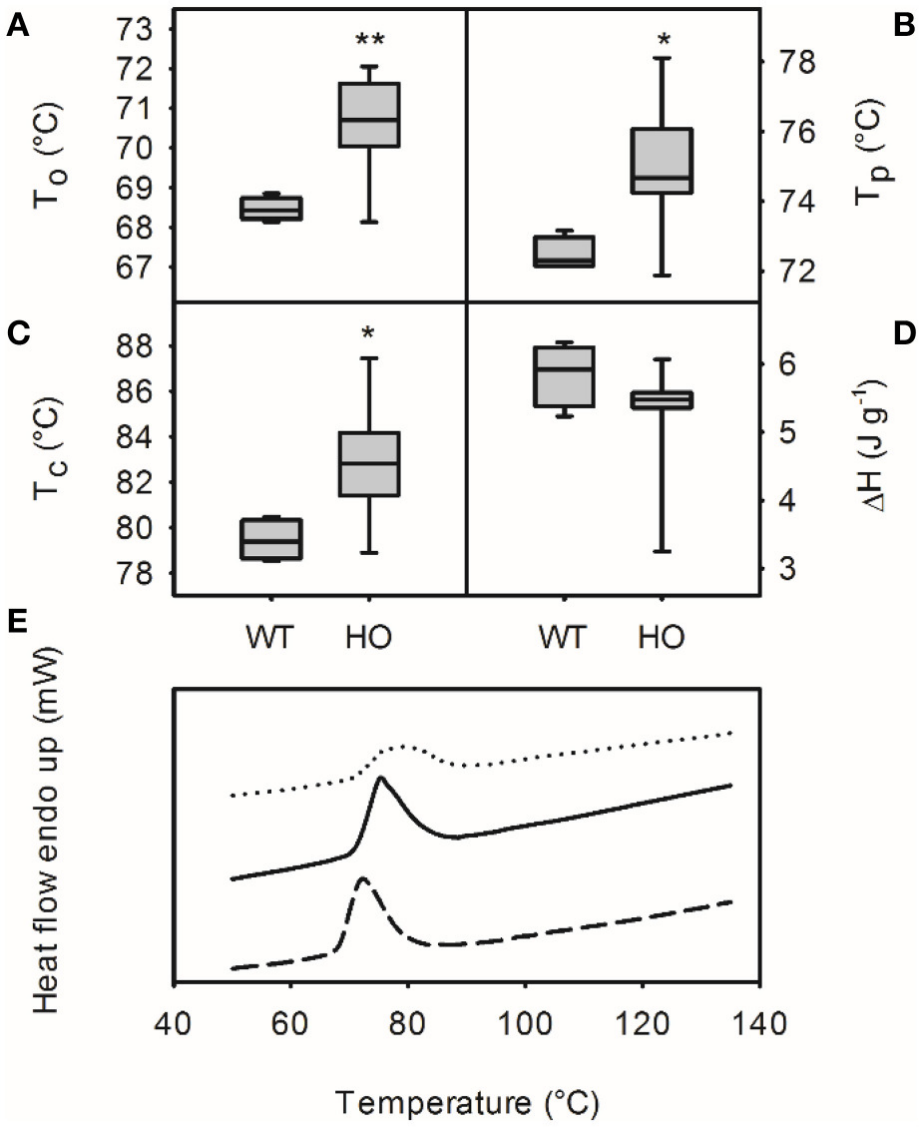
**Chemical properties of starch from wild-type (WT) and transgenic high oil (HO) potatoes by differential scanning calorimetry**. Onset temperature (T_o_; **A**), conclusion temperature (T_c_; **B**), peak temperature (T_p_; **C**) and enthalpy of transition (ΔH; **D**). Asterisks indicate significant differences between WT and HO (*t*-test: ^*^p < 0.05; ^**^p < 0.01). Representative profiles for a WT (dashed line), a medium TAG line (1.63% TAG; solid line) and the highest oil plant (3.35% DW TAG; dotted line) are also shown **(E)**.

The chemical properties of starch from wild-type and high oil potatoes were also investigated using differential scanning calorimetry (Figure 6). Starch from high oil potatoes had higher onset temperature (T_o_; mean values of 70.6 and 68.5°C, respectively; *t*-test, *p* < 0.01), end temperature (T_c_; mean values of 82.9 and 79.5°C, respectively; *t*-test, *p* < 0.05) and peak temperature (T_p_; mean values of 75.0 and 72.5°C, respectively; *t*-test, *p* < 0.05) than wild-type starch but did not have a significant different enthalpy of transition (ΔH; mean values of 5.3 and 5.8 J g^−1^, respectively). Correlation analysis identified significant relationships between starch granule size and onset temperature (*r* = −0.63, *p* < 0.05), end temperature (*r* = −0.63, *p* < 0.05) and peak temperature (*r* = −0.63, *p* < 0.05) but no correlation between apparent amylose content or TAG content and any of these chemical properties of starch. DSC profiles of starch from wild-type and medium TAG lines showed a large, sharp peak whereas starch from high TAG lines showed a lower, broader peak.

## Discussion

In addition to large reductions in starch content, high oil potatoes had such strong differences in starch chemistry that thermal and pasting properties were severely affected, especially in lines accumulating the highest levels of TAG. Large changes in the levels of other metabolites were also observed in high oil potato lines, which may affect their nutritional and processing properties. While TAG accumulation might be expected to affect potato starch and soluble sugar content through competition for carbon, the dramatic changes to nitrogen partitioning and starch structure are likely indirect effects. The high oil potato lines thus provide an interesting model to investigate different levels of metabolic and biosynthetic regulation as well as the effects of starch structure on gelatinization and pasting profiles. The potential for these potatoes as a novel source of oil or specialized crop will also be discussed.

The structure and chemistry of starch granules was investigated in detail in order to identify possible causes of the striations, altered granule shape and reduced granule size. High oil potato starch had significantly reduced amylose content. This combination of low amylose content and small, misshapen granules is surprising given that striations and a pocked surface are usually observed in high amylose mutants (Schwall et al., 2000; Hofvander et al., 2004; Andersson et al., 2006; Glaring et al., 2006) and that low amylose (GBSS antisense) potato lines have normal granule morphology (Kuipers et al., 1994). Despite the reduction in amylose content, there was no difference detected in the amount of GBSS, the enzyme responsible for amylose synthesis. Hofvander et al. (2016) also found reduced amylose content and small, irregular starch granules without any significant changes in *GBSS* expression in TAG-accumulating (*WRI1*-overexpressing) potatoes.

However, GBSS activity may be regulated through substrate availability or at the post-translational level. GBSSI produces amylose through the extension of long chain amylopectin followed by cleavage and release of the long chains. This process thus requires both high levels of substrate and physiological time to occur (Maddelein et al., 1994). The accumulation of soluble sugar could indicate a bottleneck in the accessibility of substrate required for the late amylose synthesis process allowing only the glucose-dense amylopectin molecule to be synthesized. Differences in the affinity of GBSS and soluble starch synthase for ADP-glucose could account for the altered amylose/amylopectin ratio (Clarke et al., 1999). The observed decrease in citrate in high oil potatoes may also have a detrimental effect on GBSSI activity since citrate increases GBSSI activity *in vitro* (Edwards et al., 1999). Finally, an additional explanation is that reduced apparent amylose content is simply due to reduced granule size, a correlation that has been previously, but not consistently, observed in potato (Noda et al., 2005; Dhital et al., 2011).

Starch from high oil potatoes also had reduced phosphorylation of both the C3- and C6- positions of glucose residues but the mechanism behind this change is unclear. Phosphorylation occurs mostly on the amylopectin fraction (Blennow et al., 2002) and smaller starch granules tend to have a higher phosphorous content (Neilsen et al., 1994; Noda et al., 2005). This suggests that high oil potato starch should have a higher phosphate content given the increased amylopectin content and smaller granules. Phosphorylation of starch is dependent on amylopectin branching and chain length as well as the activities of α-glucan water dikinase (GWD) and phosphoglucan water dikinase (PWD), neither of which are differentially expressed in *WRI1*-overexpressing potatoes (Hofvander et al., 2016). While no differences in amylopectin chain length distribution (enzyme substrate) were detected, branching was not quantified in this study and could still play a role. Post-translational regulation of GWD or PWD due to altered substrate (starch and soluble carbohydrate) concentrations could contribute to differential phosphorylation of starch but remains to be investigated.

The presence of more than one hilum per granule, and smaller, more numerous granules, could be due to increased activity of starch synthase IV (SSIV; AT4G18240.1) and/or plant glycogenin-like starch initiation proteins 1, 2, 5, and 6 (PGSIP; AT3G18660.2, AT1G77130.1, AT1G08990.1, AT5G18480.1, respectively) which are all upregulated at the transcript level in *WRI1*-overexpressing potatoes (Hofvander et al., 2016). SSIV is responsible for starch granule initiation in Arabidopsis with mutations in SSIV leading to fewer, larger granules (Roldán et al., 2007; Szydlowski et al., 2009). SSIV also shows high levels of starch synthase activity *in vitro* using malto-oligosaccharides as substrate, suggesting that impairment of starch synthesis might increase granule initiation (Szydlowski et al., 2009). However, no difference in the size or morphology of starch granules was observed in potatoes overexpressing *SSIV* alone (Gamez-Arjona et al., 2011). PGSIP1 may be involved in starch granule initation in Arabidopsis leaves (Chatterjee et al., 2005) so upregulation of *PGSIP* transcripts in high oil potatoes could also increase starch granule number.

Given that overexpression of *WRI1* affects only a small number of starch-related genes, it remains unclear how TAG accumulation leads to altered biosynthesis, chemistry and morphology of starch granules. While transcripts for *SSIV* and the *PGSIP*s are upregulated in *WRI1*-overexpressing potatoes, the *AGPase small subunit* transcript (AT5G48300.1) is downregulated and no other starch-related genes are affected (Hofvander et al., 2016). Starch granule size and morphology in high oil potatoes is thus likely to be regulated post-transcriptionally and may be the result of more than one factor or gene. The biosynthesis and phosphorylation of starch may be more sensitive to substrate rather than enzyme concentration. For example, AGPase is redox-regulated, activated by sucrose and glycerate-3-phosphate and inactivated by inorganic phosphate (Sowokinos, 1981; Sowokinos and Preiss, 1982; Tiessen et al., 2002). The accumulation of both sucrose (an activator) and inorganic phosphate (an inhibitor) in high oil potato lines highlights the complexity of this level of regulation, particularly with respect to intracellular metabolite pools.

Whatever the mechanism behind these changes to starch structure, they strongly reduce starch viscosity and swelling power as well as impeding gelatinization. Interestingly, the lines accumulating the highest levels of TAG had very different pasting and thermal profiles compared to both wild-type and lines accumulating only moderate levels of TAG. Starch amylose and phosphate content correlated with peak and final viscosity while granule size was significantly correlated with all pasting and thermal parameters and amylose content. Reduced granule size and reduced starch phosphate content are known to reduce the swelling power as well as the peak viscosity of starch (Wiesenborn et al., 1994; Viksø-Nielsen et al., 2001; Noda et al., 2005), which is consistent with the current study. However, none of these structural elements showed a disproportionately large change in potatoes accumulating the highest levels of TAG. Perhaps the unusual combination of low phosphorylation with small, amylopectin-rich granules has a cumulative effect on starch thermal and pasting properties.

In addition to greatly altered starch chemical and physical properties, changes in the levels of a number of metabolites were associated with TAG accumulation. The increase in nitrogen content of high oil potatoes is likely due to increased amino acid content but it is unclear exactly how lipid accumulation might lead to accumulation of free amino acids. Transgenic plants might sense the increased sucrose (available carbon) via the trehalose-6-phosphate pathway and then induce amino acid biosynthesis in an attempt to balance the carbon-to-nitrogen ratio, as occurs in Arabidopsis leaves (Figueroa et al., 2016). However, sucrose accumulation has been found to be negatively correlated with amino acid concentration and storage protein accumulation in sink organs (Muller-Rober et al., 1992; Roessner-Tunali et al., 2003). Amino acid accumulation could also be linked to *WRI1* expression, which leads to altered amino acid-related gene expression in leaves, including the upregulation of asparagine synthetase and glutamate synthase transcripts (Grimberg et al., 2015). However, this upregulation of asparagine synthetase in leaves is inconsistent with the observed decrease in asparagine in potato tubers and this gene was not identified as differentially expressed in *WRI1*-overexpressing potatoes (Hofvander et al., 2016). Whether these amino acids are transported to or synthesized in potato tubers is also unclear. Asparagine, for example, is mainly synthesized in potato tubers whereas glutamate, glutamine, serine and alanine tend to be transported from leaves along with some aspartate, GABA, glycine, phenylalanine, proline, threonine, and valine (Muttucumaru et al., 2014a). Labeling studies and measurements of sap metabolites could be used to determine the source of these amino acids.

Other notable differences in soluble metabolite accumulation include increased fumarate and malate but decreased citrate in high oil potato lines, which could be related to changes in the TCA cycle and increased demand for substrates for fatty acid synthesis. The concomitant decrease in citrate could be indicative of the strong pull of the fatty acid synthesis pathways as a result of *WRI1* expression. The observed increase in inorganic phosphate may be due to altered demand for ATP for lipid and/or starch biosynthetic pathways. Transcriptome analysis of the highest oil potato lines from this study is also underway and will shed further light on the changes to gene expression underlying the observed changes metabolite pools.

The successful metabolic engineering of novel crops requires the parent plant to retain its nutritional and commercial value in addition to the introduction of the desired trait. Leaf oil crops have the potential to become dual sources of oil and seed while oil-accumulating potatoes could become sources of oil and starch. One potato line described in this study already accumulates sufficient oil (3.3% TAG by dry weight) to deliver yields comparable to cotton seed, potentially making it a commercially feasible source of oil (Liu et al., 2017). However, the 30–40% reduction in starch content observed in this line would limit its use as a source of starch, as would the dramatic changes in starch chemical and physical properties. While the low viscosity of high oil potato starch may find applications in the food industry, changes to amylose are small compared to amylose-free potato lines and higher not lower phosphate content is generally desirable (Jobling, 2004). These trade-offs, along with any potential yield penalty, need further investigation. Further manipulation of carbon partitioning, starch synthesis and/or degradation could help overcome some of these limitations.

An alternative approach would be to use potatoes accumulating medium levels of TAG for novel products or added nutrition. The starch content and quality of these lines is close to wild-type and TAG accumulation correlates with changes to other metabolites that may give nutritional or processing benefits. For example, the high oil potatoes are notably low in asparagine, normally the most common amino acid in potato (Golan-Goldhirsh, 1986; Koch et al., 2003), but which can react with sugars during frying to produce acrylamide, a potential carcinogen (Chawla et al., 2012). However, the relationship between asparagine content and acrylamide formation is complex and the high sugar content of these potatoes could also be a problem during frying (Muttucumaru et al., 2014b). Nevertheless, high oil potatoes are more energy dense and have elevated levels of nutrients including most proteinogenic amino acids, nicotinic acid (niacin/vitamin B3) and ascorbic acid (vitamin C) making them interesting candidates for high value food products.

In summary, metabolic engineering of potato tubers to accumulate high levels of TAG also resulted in potatoes with altered nutritional profiles, starch structure and quality. Starch granule size, morphology, chemical and physical properties were particularly affected, which may have implications for downstream applications and processing. Future manipulations of carbohydrate pathways as well as source/sink relationships could further improve potato, oil, and/or starch yields. Such strategies have the potential to produce novel crops with benefits to biotechnology and the food industry as well as human nutrition.

## Author contributions

MM conceived of and performed experiments, analyzed data and wrote the manuscript. JP, SO, OL, DY, and NS performed experiments and analyzed data. QL provided plant material. FP and SS participated in the design and coordination of the study. JR conceived of experiments, analyzed data, contributed to the manuscript and participated in the design and coordination of the study. All authors read and approved the manuscript.

## Funding

This research was funded by CSIRO Agriculture and Food.

### Conflict of interest statement

The authors declare that the research was conducted in the absence of any commercial or financial relationships that could be construed as a potential conflict of interest.

## Abbreviations

DW: dry weight
GBSS: granule-bound starch synthase
GC: gas chromatography
MS: mass spectrometry
TAG: triacylglycerol
HO: high oil
WT: wild-type.
