# Supplementary material for: Oil Accumulation in Transgenic Potato Tubers Alters Starch Quality and Nutritional Profile
Source: Front Plant Sci. 2017 Apr 12;8:554. doi: 10.3389/fpls.2017.00554 (PMC5388768; doi:10.3389/fpls.2017.00554)
Supplement: Supplementary file 1 [file DataSheet1.DOCX]

**Mitchell et al. Supplemental Tables and Figures**

Supplemental Table 1. Correlation coefficients for potato carbon and nitrogen content in wild-type (WT) and high oil (HO) potatoes. Correlation coefficients and p-values (** p < 0.01; * p < 0.05) were calculated using Microsoft Excel. CHO (carbohydrate) is the sum of starch and soluble sugars.

|  | ***TAG*** | ***energy density*** | ***total C*** | ***CHO*** | ***starch*** | ***sugars*** | ***cellulose*** | ***N*** | ***soluble protein*** |
| --- | --- | --- | --- | --- | --- | --- | --- | --- | --- |
| **energy density** | 0.98** |  |  |  |  |  |  |  |  |
| **total C** | -0.24 | -0.09 |  |  |  |  |  |  |  |
| **CHO** | -0.75** | -0.60* | 0.60* |  |  |  |  |  |  |
| **starch** | -0.83** | -0.69* | 0.57 | 0.97** |  |  |  |  |  |
| **sugars** | 0.85** | 0.78** | -0.42 | -0.77** | -0.89** |  |  |  |  |
| **cellulose** | 0.53 | 0.47 | -0.57 | -0.54 | -0.52 | 0.41 |  |  |  |
| **N** | 0.94** | 0.88** | -0.41 | -0.85** | -0.91** | 0.89** | 0.54 |  |  |
| **soluble protein** | 0.19 | 0.19 | -0.13 | -0.15 | -0.09 | -0.02 | 0.39 | 0.33 |  |
| **C:N** | -0.92** | -0.86** | 0.39 | 0.81** | 0.86** | -0.82** | -0.48 | -0.98** | -0.39 |

Supplemental Table 2. Correlation coefficients for properties of starch granules from wild-type (WT) and high oil (HO) potatoes. Correlation coefficients and p-values (** p < 0.01; * p < 0.05) were calculated using Microsoft Excel.

|  | ***% TAG*** | ***Granule size*** | ***% amylose*** | ***Glc-3-P*** | ***Glc-6-P*** |
| --- | --- | --- | --- | --- | --- |
| **Granule size** | -0.76** |  |  |  |  |
| **% amylose** | -0.79** | 0.80** |  |  |  |
| **Glc-3-P** | -0.76** | 0.86** | 0.76** |  |  |
| **Glc-6-P** | -0.91** | 0.78** | 0.64* | 0.85** |  |
| **Peak viscosity** | -0.97** | 0.78** | 0.79** | 0.76** | 0.92** |
| **Hold viscosity** | 0.30 | -0.72** | -0.33 | -0.48 | -0.43 |
| **Final viscosity** | 0.82** | -0.70* | -0.63* | -0.63* | -0.78** |
| **Onset temp (To)** | 0.03 | -0.63* | -0.33 | -0.51 | -0.19 |
| **End temperature (Tc)** | 0.11 | -0.63* | -0.31 | -0.57 | -0.26 |
| **Peak temperature (Tp)** | 0.09 | -0.63* | -0.26 | -0.55 | -0.28 |
| **Enthalpy (ΔH)** | -0.35 | 0.42 | 0.22 | 0.59* | 0.51 |
| **Swelling power (B/S_M_)** | -0.23 | -0.20 | 0.19 | -0.10 | 0.04 |


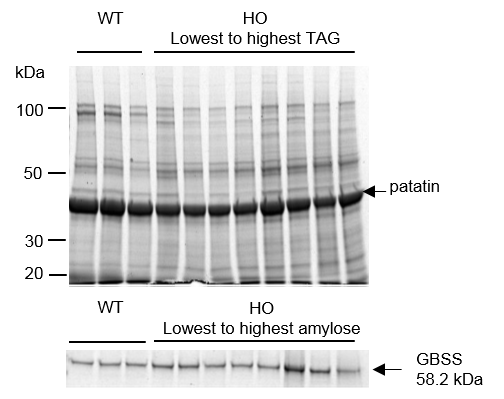


Supplemental Figure 1. SDS-PAGE analysis of total protein from WT and high oil potato lines normalised by potato dry weight. Starch-associated granule-bound starch synthase (GBSS) protein normalised by starch fresh weight is also shown.

***
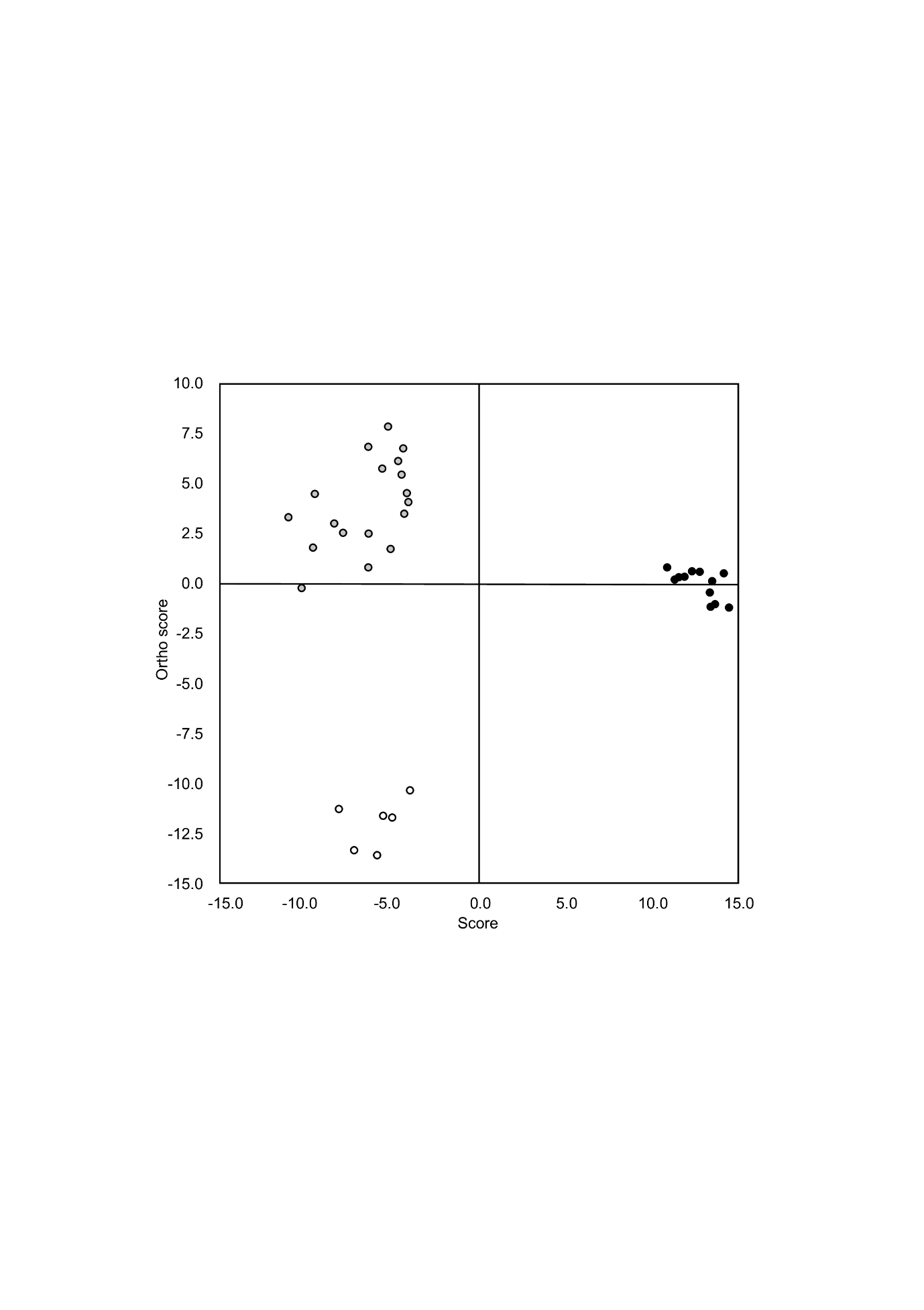
***

Supplemental Figure 2. Orthogonal partial least squares analysis of metabolites from wild-type and high oil potatoes. Wild-type (black circles), medium TAG (grey circles) and high TAG (white circles) potato lines are clustered.

Supplemental Figure 3. Chain length distribution of amylopectin, digested with isoamylase or β-amylase, from wild-type (red) and high oil (black) potatoes. Curves shows the mean of two technical replicates for each line (four wild-type and eight high oil).

**Supplemental Methods**

Soluble protein was extracted in buffer (Tris pH 7.5, 10 mM MgCl_2_) and quantified by Bradford assay (Bradford, 1976). Potato flour (total protein), soluble protein or isolated starch (starch-bound protein) was added to loading buffer, boiled for 5 min and the supernatant was run on an SDS-PAGE gel (Laemmli, 1970). Proteins were stained using EZBlue^TM^ Gel Staining Reagent (Sigma-Aldrich). Gels were scanned and the bands quantified using TotalLab Quant software (TotalLab, Newcastle upon Tyne, UK).
